# Application of off‐line image processing for optimization in chest computed radiography using a low cost system

**DOI:** 10.1120/jacmp.v16i2.4774

**Published:** 2015-03-08

**Authors:** Wilbroad E. Muhogora, Peter Msaki, Renato Padovani

**Affiliations:** ^1^ Ionizing Radiation Department Tanzania Atomic Energy Commission Arusha Tanzania; ^2^ Physics Department University of Dar es Salaam Dar es Salaam Tanzania; ^3^ Fisica Sanitaria, Ospedale‐Universitario Udine Italy

**Keywords:** computed radiography, digital image, off‐line image processing, image quality criteria

## Abstract

The objective of this study was to improve the visibility of anatomical details by applying off‐line postimage processing in chest computed radiography (CR). Four spatial domain‐based external image processing techniques were developed by using MATLAB software version 7.0.0.19920 (R14) and image processing tools. The developed techniques were implemented to sample images and their visual appearances confirmed by two consultant radiologists to be clinically adequate. The techniques were then applied to 200 chest clinical images and randomized with other 100 images previously processed online. These 300 images were presented to three experienced radiologists for image quality assessment using standard quality criteria. The mean and ranges of the average scores for three radiologists were characterized for each of the developed technique and imaging system. The Mann‐Whitney U‐test was used to test the difference of details visibility between the images processed using each of the developed techniques and the corresponding images processed using default algorithms. The results show that the visibility of anatomical features improved significantly (0.005≤p≤0.02) with combinations of intensity values adjustment and/or spatial linear filtering techniques for images acquired using 60≤kVp≤70. However, there was no improvement for images acquired using 102≤kVp≤107 (0.127≤p≤0.48). In conclusion, the use of external image processing for optimization can be effective in chest CR, but should be implemented in consultations with the radiologists.

PACS number: 87.59.−e, 87.59.−B, 87.59.−bd

## I. INTRODUCTION

Many anatomical structures of clinical interest in computed radiographs are known to have low signal‐to‐noise ratio (SNR) and low contrast‐to‐noise ratio (CNR). This often causes many image processing algorithms to perform poorly with a consequence of ineffective diagnosis.^(1)^ The emerging of low cost CR systems generation has brought another challenge of being suspicious to posses even lower SNR and CNR than conventional CR systems, consequently prompting the use of relatively higher doses to patients.^(2)^ A number of studies have therefore been devoted to investigate on how best to clinically customize image processing algorithms.^3^, ^4^, ^5^, ^6^ Of particular interest is the use of external postimage processing to complement online processing. In practice this task is implemented by radiographers who adjust contrast, brightness, and sharpness or noise levels before presenting the images to radiologists. However, experience has shown that there is considerable variation of image qualities from different algorithms, as reported by radiologists.^5^, ^6^ This has partly been attributed to low CNR and SNR properties, as well as the lack of knowledge on the implemented online image processing. Another possible cause of such image quality variations is the subjective nature inherent in the postimage processing as performed by radiographers.^(5)^

In a previous study, it has been observed that, although the low cost CR system (LCCS) can display anatomical details comparably to conventional CR system (CCS), it is associated with higher patient dose by a factor of up to 2.5 for similar sized patients.^(2)^ In that study, the relative comparison between the two systems was done since the systems were being operated at similar high tube potentials (high kVps). However, LCCS was also being operated at low tube potentials (low kVps), which motivated further investigations to compare the image quality and patient dose for LCCS at high and low kVps. Since the results from the earlier study^(2)^ had shown inadequate image quality with relatively higher average patient dose at high kVps, it was reasonably inferred that the image quality at low kVps could also be inadequate. This view motivated an attempt to improve the visibility of anatomical details by applying external postimage processing on chest computed radiographs acquired using both low and high kVps. The objective of this study was, therefore, to develop quantitative spatial domain‐based external image processing techniques and evaluate their potentials for the improvement of anatomical visibility on studied low cost CR system.

## II. MATERIALS AND METHODS

### A. Imaging system

The imaging system that was employed during this study has also been used earlier.^(2)^ It was a low cost Philips PCR Campano (Philips Healthcare, Eindhoven, The Netherlands) (herein after called Philips PCR) and was being operated with standard Philips Bucky Duo Diagnostic radiographic equipment (Philips Medical Systems, Hamburg, Germany), which had previously passed a quality control check. The CR system used to read the image plates was a Fuji Type C (Fuji Photo Film Company Limited, Tokyo, Japan) using standard (300 speed) postprocessing algorithm. The final processed image of the CR system was reduced to 8‐bit in Joint Photo Graphic Experts Group (JPEG) format. This format was set by vendors to reduce size of images as images backup is done using compact disks.

### B. Collection of chest radiographs

The study was approved by the authority of the hospital and informed consent obtained from each patient. The chest PA images were collected in two weeks at 60–70 kVp (mean 64 kVp) and 102–109 kVp (mean 107 kVp). The selection of tube potential ranges was motivated by the preference of reporting radiologist at the hospital. Table 1 summarizes the imaging parameters of Philips PCR that were used during the collection of images. The estimation of radiation doses imparted to patients for such parameters under this study is given in Table 2. It can be seen that the dose to patients examined using low potentials was on the average 60% less that the dose for patients of nearly similar sizes examined using high tube potentials. This implies that the dose to the detector in the earlier case was lower than for the latter case.

**Table 1:** Imaging parameters on Philips PCR system during the collection of chest images. Data on high kVp are reproduced from previous study.^(2)^

*Equipment*	*Image Processing Software/ Radiographic Technique*	
Philips Campano CR system	standard/high tube potential	standard/low tube potential
Philips duo‐diagnost X‐ray equipment		
Tube potential (kVp)	range :102–109; mean: 107	range: 60–70; mean: 64
Half value layer (mm Al)	range: 4.2–4.6	range: 2.4–2.8
Tube current‐time product (mAs)	range: 3–4; mean: 3.52	range: 5–7; mean: 6.2
Source–image distance (cm)	155	155
Grid ratio/frequency	$12/36\;{\text{cm}}^{-1}$	$12/36\;{\text{cm}}^{-1}$

**Table 2:** Patient characteristics and the estimated entrance surface dose (ESD) during image collection. Low/high kVp refers to the X‐ray technique used. Data on high kVp are reproduced from previous study.^(2)^

*Parameter*		*Thickness (cm)*	*Weight (kg)*	*Tube Voltage (kVp)*	*Tube Charge (mAs)*	*ESAK (μGy)*
Low kVp	range	17–26	63–85	60–70	5–7	166–208
	mean	21	72	64	6.2	180
High kVp	range	20–24	60–82	102–109	3–4	259–367
	mean	22	70.3	107	3.5	302

### C. Development of image processing algorithms

**Figure 1:**
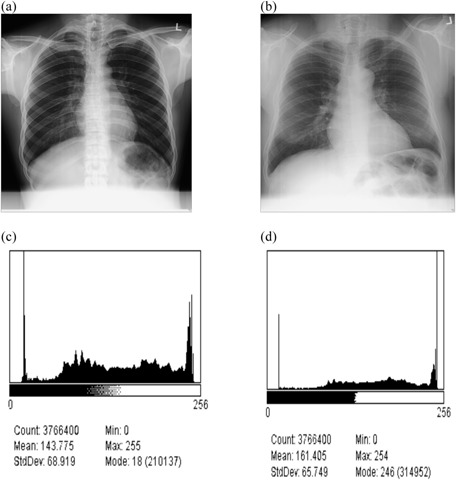
An example of chest radiograph acquired on Philips Campano at (a) 70 kVp technique and (b) 107 kVp technique, and their respective histograms (c) and (d).

First, the histograms of all images were analyzed using standard image processing and analysis freeware software Image J.^7^, ^8^ The sample histogram distributions are shown in Fig. 1. According to Gonzalez et al.,^(9)^ if the histogram components are concentrated on the low side of grey scale, then the image area is dark. A histogram biased on high side of gray scale implies a bright image region. Also it is suggested that a low contrast region is characterized by a narrow histogram centered in the middle of the gray scale area. Furthermore, histogram curves with a broad range of the gray scale with almost uniform distribution of pixels represent high contrast region.^(9)^ Such analysis suggests the image in Fig. 1(a) with histogram concentrated on lower intensity range extending to the middle to be dark and low contrast characteristic. The image in Fig. 1(b) with relatively uniform histogram exhibits high‐contrast properties. From the analysis of the histograms patterns, suggested background subtraction, intensity values adjustment, histogram equalization, and spatial linear filtering were hypothesized to be potential external image processing techniques.

Second, the sequence followed in developing the algorithms using MATLAB software version 7.0.0.19920 (R14) (MathWorks, Natick, MA) and the image processing toolbox was as follows:

1. The background level of each sample clinical radiograph was assumed to be mainly due to photon statistics and therefore additive noise. Such noise, which is also known to be independent of image coordinates, was estimated from each of the uint 8 image in the whole range [0,255] with double precision command. It is known that the latter command has no effect, even if this had been implemented to the original image or not. The background was then subtracted from the image to create a uniform background image. Since after background subtraction the image tends to be dark,^(9)^ the contrast of the processed image was adjusted by saturating the data at both low and high intensities of image. Experimentation using 0.5% to 5% saturation showed that 1% had no visible effect on the natural appearance of radiographs and, therefore, was used in this study.
2. After saturation process, the adjustment of processed image to fill the dynamic energy range of interest was performed by stretching the intensity values of the images (i.e., intensity values adjustment). Because of lack of precise information of previously implemented online processing algorithms, the entire energy range, which is the default value, was specified.
3. The filtering technique was experimented with an aim to smooth images (i.e., reduce noise). In this technique, the interest was to find a suitable coefficient (filter mask or kernel) which, if multiplied by each pixel and summing the results, the response of each point could be obtained. The process was then repeated for every point in the image, thus improving detail visibility. In order to achieve this, linear shift invariant operation was adopted^1^, ^9^, ^13^ which means that every pixel is replaced with a linear combination of its neighbors. The image processing tool box requires the specification filtering mode (convolution or correlation), boundary conditions (padding, replica, symmetric or circular), and the size option (full or same). A convolution filtering mode, which is rotated by 180° prior to its application, was applied since the interest was to smooth the images to reduce noise and not match operations achieved by correlation.^(9)^ In addition, the convolution operation is associative, which could fit well the situation of inexact knowledge of the previously implemented online image processing. Further, the default boundary condition (i.e., padding=0) was applied due to insufficient information of image processing algorithms previously applied to the images. For similar reasons, the default size option (i.e., “same”) was used. This means that the output image was of the same size as the input image. The suitability of different values of filter mask (w) on sample images ranging from 0.2×0.2 to 10×10 was investigated to establish an optimum filter size. Filter values of 0.3×0.3 and 0.4×0.4 produced good visual images.
  The size of 0.3×0.3 was selected for intended image processing since the smaller the filter size, the better details visibility.

Third, various combinations utilizing the four image processing techniques developed above were studied. This followed from the fact that most of clinical radiographs are usually characterized by low contrast, unsharpness, and noise. Therefore, a total of four off‐line postimage processing techniques were developed using different combinations of techniques. These were background subtraction combined with intensity values adjustment, intensity values adjustment combined with histogram equalization, intensity values adjustment combined with linear spatial filtering, and linear spatial filtering alone.

Each of the developed techniques was implemented on 50 previously collected clinical chest radiographs. This implies that for four developed image processing techniques, a total of 200 externally processed images were obtained. These 200 images were randomized with additional 100 processed images using default algorithms (i.e., online postimage processing). These 300 images were presented to three experienced radiologists for independent image quality assessment on conventional monitor model XPS 7100 DELL computer (Round Rock, TX), since reporting display monitors are currently inexistent. The experiences of radiologists ranged from 10 to 20 years; one was working at University Hospital, while the rest were working at the National Hospital. The observers were requested to score each image against the six quality criteria shown in Table 3,^(11)^ and to indicate their level confidence on the basis of the 5‐point scale shown in Table 4.^(12)^

The scores of each criterion from the four radiologists were averaged for each image. The overall average score for each criterion was then obtained for all images. The analysis of data was performed under four groups of the image quality criteria, which were group 1 (criteria a–f), group 2 (criteria a–d), group 3 (criterion e), and group 4 (criterion f), per Table 4. The average raw scores of four observers for all images for each imaging technique were tested for significance with the Mann‐Whitney U‐test. The difference between the additionally external processed set of images and those processed online alone was considered significant if the probability *p* was less than 0.05 at 95% confidence level. P was obtained as a two‐sided probability using normal approximation.

**Table 3:** Six image quality criteria used to evaluate the chest PA images.^(11)^

*Criteria*	*Description of Criteria*
a	Performed at full inspiration (as assessed by the position of the ribs above the diaphragm (either 6 anteriorly or 10 posteriorly) and with suspended respiration
b	Symmetrical imaging of the thorax as shown by the central position of a spinous process between the medical ends of the clavicles
c	Medical border of the scapulae to be outside the lung fields
d	Visualization of the whole lung including the costophrenic angles
e	Clear visualization of the lung structure and vascular pattern throughout the lung fields including the retrocardiac area
f	Clear delineation of vertebral disc spaces

**Table 4:** Criteria for confidence rating for each anatomical detail.^(12)^

*Rating*	*Score*
Confident that the criterion was fulfilled	5
Somehow confident that the criterion is fulfilled	4
Indecisive whether the criterion is fulfilled or not	3
Somehow confident that the criterion is not fulfilled	2
Confidence that the criterion is not fulfilled	1

## III. RESULTS

### A. Chest radiographs after implementation of external postimage processing

2, 5 show the radiographs acquired using low potential (low kVp radiographs) before and after implementation of external postimage processing algorithms. Visual improvement as a result of external image processing can be observed in 2, 3, where respective background subtraction in combination with intensity values adjustment and intensity values adjustment with histogram equalization techniques were applied.

This suggests that the original images had nonuniform background, and/or dark or low contrast characteristic. It is also observed that in some cases, where intensity values adjustment and linear spatial filtering techniques were applied (4, 5), the visual appearances of images after the application of external image processing were slightly darker. This probably suggests that the noise content of the original images was at optimum levels, given the fact that intensity values adjustment showed adequate appearance with regard to 2, 3.

**Figure 2:**
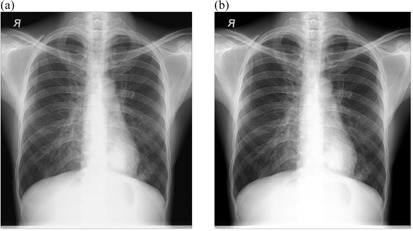
Low kVp chest radiograph: (a) original image and (b) processed image by background subtraction and pixel adjustment.

**Figure 3:**
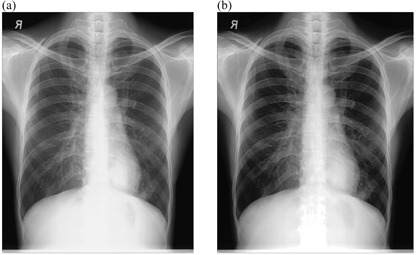
Low kVp chest radiograph: (a) original image and (b) processed image by pixel adjustment and histogram equalization.

**Figure 4:**
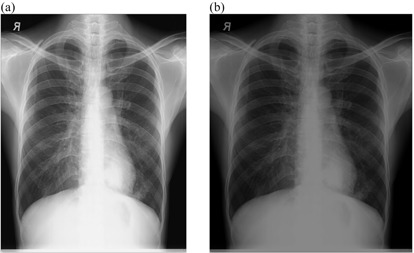
Low kVp chest radiograph: (a) original image and (b) processed image by pixel adjustment and linear spatial filtering.

**Figure 5:**
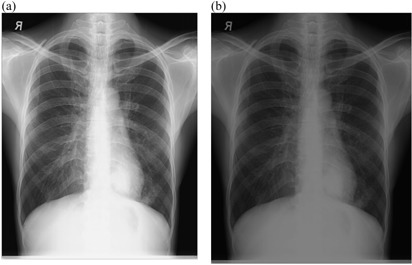
Low kVp chest radiograph: (a) original image and (b) processed image by linear spatial filtering.

**Figure 6:**
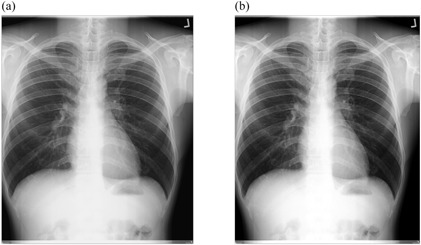
High kVp chest radiograph: (a) original image and (b) processed image by background subtraction and pixel adjustment.

**Figure 7:**
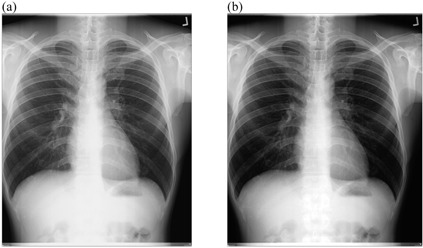
High kVp chest radiograph: (a) original image and (b) processed image by pixel adjustment and histogram equalization.

**Figure 8:**
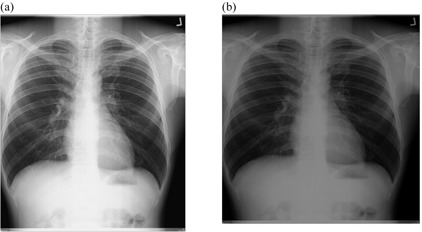
High kVp chest radiograph: (a) original image and (b) processed image by pixel adjustment and linear spatial filtering.

**Figure 9:**
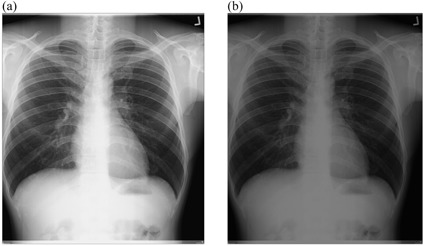
High kVp chest radiograph: (a) original image and (b) processed image by linear spatial filtering alone.

6, 9 show the radiographs acquired using high potential (high kVp radiographs) before and after the implementation of external post image processing algorithms. There was no visual difference between the original and processing images with respect to background subtraction with intensity values adjustment technique (Fig. 6). However, the application of intensity values adjustment with histogram equalization (Fig. 7), intensity values adjustment with linear spatial filtering (Fig. 8), or linear spatial filtering techniques (Fig. 9) resulted to darker images. Since the radiographs were acquired at high kVps, the images were not relatively noisy, dark or low contrast characteristic (Fig. 1) that resulted to inadequate visual appearance after the application of such processing techniques.

### B. Evaluation of clinical image quality by radiologists

The results of image quality assessment before the application of developed image processing techniques are presented in Table 5. It is clear that criteria (a) to (d) reflect the technique used (e.g., positioning, collimation, centering) and therefore they were not expected to change with subsequent external image processing. Therefore, from now onwards any “image quality improvement” will refer to the image quality assessment with regard to criteria (e) and (f). The criteria are respective “clear visualization of the lung structure and vascular pattern throughout the lung fields including the retrocardiac area” and “clear delineation of vertebral disc spaces” (Table 3). Table 6 presents the results of image quality assessment after the application of background subtraction and pixel adjustment technique. The results showed image quality improvement only for criterion “e” on low tube potential (low kVp) technique (p=0.005).

Table 7 presents the results of image quality assessment after the implementation of intensity values adjustment and histogram equalization technique. This time the image quality improvement was observed for criterion (f), but again of low kVp technique. The results of image quality assessment after the implementation of pixel adjustment and linear spatial filtering technique are presented in Table 8. The use of these techniques resulted in the improvement of criteria (e) (p=0.03) and (f) (p=0.047) for low kVp technique. Similar results were obtained with the application of linear spatial filtering technique alone (Table 9). In this case the image quality improved on criteria (e) (p=0.03) and (f) (p=0.025).

**Table 5:** Consolidated image quality criteria scores before external image processing

*Image Quality Criteria (see* Table 3)	*Low Tube Potential*		*High Tube Potential*	
	*Range*	*Mean*	*Range*	*Mean*
a–d	3.75−5	4.37	3.44−5	4.52
e	3−5	4.65	4−5	4.87
f	1−4.25	3.37	3−4.25	3.77

**Table 6:** Consolidated image quality criteria scores after external processing using background subtraction and pixel adjustment technique

*Image Quality Criteria (see* Table 3)	*Low Tube Potential*		*p‐value*	*High Tube Potential*		*p‐value*
	*Range*	*Mean*		*Range*	*Mean*	
a–d	3.56–5	4.32	0.352	3.75–5	4.6	0.397
e	3–5	4.93	0.005	–	5	0.212
f	1–5	3.33	0.397	2.25–4.75	3.77	0.4801

Dash (–)=no data range.

**Table 7:** Consolidated image quality criteria scores after external processing using pixel adjustment and histogram equalization technique

*Image Quality Criteria (see* Table 3)	*Low Tube Potential*		*p‐value*	*High Tube Potential*		*p‐value*
	*Range*	*Mean*		*Range*	*Mean*	
a–d	3.69–5	4.45	0.41	3.5–5	4.46	0.468
e	4−5	4.87	0.142	–	5	0.212
f	3–4.75	4.03	0.076	3–4.75	4	0.142

Dash (–)=no data range.

**Table 8:** Consolidated image quality criteria scores after external processing using pixel adjustment and linear spatial filtering technique

*Image Quality Criteria (see* Table 3)	*Low Tube Potential*		*p‐value*	*High Tube Potential*		*p‐value*
	*Range*	*Mean*		*Range*	*Mean*	
a–d	3.69–5	4.37	0.468	3.06–5‐5	4.43	0.409
e	–	5	0.03	4.75–5	4.97	0.284
f	2.25–4.75	3.95	0.047	2−4–75	4	0.127

Dash (–)=no data range.

**Table 9:** Consolidated image quality criteria scores after external processing using linear spatial filtering technique alone

*Image Quality Criteria (see* Table 3)	*Low Tube Potential*		*p‐value*	*High Tube Potential*		*p‐value*
	*Range*	*Mean*		*Range*	*Mean*	
a–d	3.56–5	4.43	0.444	3.31–5	4.43	0.367
e	–	5	0.03	4–5	4.77	0.298
f	3–5	3.67	0.025	2–4.75	3.78	0.425

Dash (–)=no data range.

## IV. DISCUSSION

Digital image processing is mainly intended to process an image so that the result is more suited to specific application than the original image.^9^, ^13^ For this reason, it is not expected that any image processing technique will be universal for all examinations or even for same type of examination for different patients. In this study, four external image processing techniques were developed to test clinical radiographs. The change in image quality was evaluated on the basis of simple quality criteria developed by a group of radiologists in Europe.^(14)^ A valid assumption of these criteria is that, if the normal anatomy can be faithfully reproduced in image, then the pathologies will also be visualized.^(14)^

Positive change in image quality was observed with the application of background subtraction in combination with intensity values adjustment (p=0.005) (Fig. 2, Table 6), as well as with the use of intensity values adjustment in combination with linear filtering techniques (p=0.03, p=0.047) for low kVp radiographs (Fig 4, Table 8). Image quality improvement for low kVp radiographs was also recorded with the application of linear spatial filtering technique alone (p=0.03, p=0.025) (Fig. 5, Table 9). The significance levels can be summarized as 0.005≤p≤0.02 for images acquired using 60≤kVp≤70. The improvement in image quality for these radiographs shows that the developed algorithms are efficient For high kVp radiographs, none of developed technique could be effective (6, 9), the significance level of which can be summarized as 0.127≤p≤0.48 for images acquired using 102≤kVp≤107. This has been explained on the basis that high kVp radiographs, in this study also obtained with a higher dose to the CR plate, are relatively less noisy, less dark, and with high contrast for the developed technique to be effective. The background subtraction usually improves contrast by making it uniform throughout the image.^(10)^ Intensity values adjustment or histogram equalization modifies the dynamic range of the image also resulting in improved contrast.^9^, ^10^, ^13^, ^15^ Linear filtering reduces the content of noise in the image thus promoting good visibility of anatomical details.^3^, ^9^, ^13^, ^15^

It is clear that the developed techniques in their present forms cannot be applied in clinical routines unless additional information technology work on programming the algorithms is done. However, the need of radiographers to consult radiologists during optimization is a positive experience that can be applied from this study. Such consultations should preferably be done after the commissioning of equipment and periodically when the need arises. This study has demonstrated that the adjustment of contrast, brightness, sharpness or noise levels that are routinely performed by radiographers may not always optimal.

Despite of the usefulness of this study, there are also possible limitations. First, the postimage processing was done in 8 bit, which is associated with some information loss when 16 bit images are changed to 8 bit. The processing should have been be done in 16 bit, but the low cost CR software does not allow such transformations. Second, this study is mainly based on histogram analysis, implying that the results may be limited to the radiographic study positioning and technique that influence the histogram shape.^(15)^ Third, the fact that the high kVp images were acquired at a much higher dose to CR plate than the low kVp images, this implies that the two sets of images were not comparable le in term of noise level. This aspect limits the comparison because the difference is not only on the technique (low and high kV), but also and mainly on dose level at the detector. Fourth, the testing of the developed techniques was done on radiographs obtained on average sized patients although proportionate results are expected if the sizes of the patient change. Fifth, the assessment of image quality was done on conventional display monitors, which usually are limited in brightness. Better results would be expected if reporting monitors (which are currently inexistent in the country) were used.

Despite these practical limitations, this study is another demonstration of the usefulness of the collaboration between radiologists and radiographers in utilizing the digital advantage to improve patient dose management.

## V. CONCLUSIONS

External image processing is a useful tool to complement online image processing in computed radiography. However, the implementation of these techniques is often subjective, thus leading to image quality variations that can affect diagnosis. In an attempt to reduce this subjectivity, four spatial domain‐based quantitative external image processing techniques were developed by manipulating contrast, unsharpness, and noise variables. Out of these, three techniques resulted in image quality improvement according to the opinion of three experienced radiologists. The techniques were background subtraction with intensity values adjustment, intensity values adjustment with linear spatial filtering, and linear spatial filtering alone. In conclusion, the use of external image processing for optimization can be effective in chest CR in improving details visualizations thus promoting good patient dose management. However, the prerequisite to this is collaboration between the radiographers and the radiologists when implementing similar image adjustments in clinical situation.

## ACKNOWLEDGMENTS

This study was performed under the joint financial support of ICTP (International Centre for Theoretical Physics) and IAEA (International Atomic Energy Agency), for which the authors are grateful. The authors also wish to thank three radiologists (Dr Ramadhan Kazema, Dr. Joseph Kimaro and Dr. Bakari Changale) for their assistance in assessing the quality of radiographs during this study.
